# Supplementary material for: What Determines Forest Farmers’ Participation in Afforestation Programs? Empirical Evidence from a Population-Based Census Survey
Source: Int J Environ Res Public Health. 2020 Jun 3;17(11):3962. doi: 10.3390/ijerph17113962 (PMC7312513; doi:10.3390/ijerph17113962)
Supplement: Supplementary file 1 [file ijerph-17-03962-s001.pdf]

Data Briefly and Computer Programs

- **Access to Census Survey**

Due to privacy concerns, the census surveys with information on individual forest farm households are not publicly available. Readers interested in this dataset should send an application form to the Directorate-General of Budget, Accounting, and Statistics of the Executive Yuan. Available at https://eng.dgbas.gov.tw/mp.asp?mp=2

- **Computer Program**

```
//** Stata Code **//
use  "F:\data_forest\data99104_farmland_s.dta"
global xvar age male elementary junior senior college under15_m under15_w
above15_m above15_w r_hh_onforest hireworker total_land1 r_selfland d_2015
sum mnl $xvar
sum mnl $xvar if mnl==0
sum mnl $xvar if mnl==1
sum mnl $xvar if mnl==2
sum mnl $xvar if mnl==3
mlogit mnl $xvar
  margins, dydx(*) predict(outcome(1))
  margins, dydx(*) predict(outcome(2))
  margins, dydx(*) predict(outcome(3))
test [1=2=3]
```
